# Ultrafine Particles Cross Cellular Membranes by Nonphagocytic Mechanisms in Lungs and in Cultured Cells

**DOI:** 10.1289/ehp.8006

**Published:** 2005-05-26

**Authors:** Marianne Geiser, Barbara Rothen-Rutishauser, Nadine Kapp, Samuel Schürch, Wolfgang Kreyling, Holger Schulz, Manuela Semmler, Vinzenz Im Hof, Joachim Heyder, Peter Gehr

**Affiliations:** 1 Institute for Anatomy, University of Bern, Bern, Switzerland; 2 Department of Physiology and Biophysics, Faculty of Medicine, The University of Calgary, Calgary, Alberta, Canada; 3 GSF-National Research Center for Environment and Health, Institute for Inhalation Biology, Neuherberg/Munich, Germany; 4 Institute of Pathophysiology, University of Bern, Bern, Switzerland

**Keywords:** aerosol, erythrocytes, lungs, macrophages, microscopy, nanoparticles, rats, surfactant

## Abstract

High concentrations of airborne particles have been associated with increased pulmonary and cardiovascular mortality, with indications of a specific toxicologic role for ultrafine particles (UFPs; particles < 0.1 μm). Within hours after the respiratory system is exposed to UFPs, the UFPs may appear in many compartments of the body, including the liver, heart, and nervous system. To date, the mechanisms by which UFPs penetrate boundary membranes and the distribution of UFPs within tissue compartments of their primary and secondary target organs are largely unknown. We combined different experimental approaches to study the distribution of UFPs in lungs and their uptake by cells. In the *in vivo* experiments, rats inhaled an ultrafine titanium dioxide aerosol of 22 nm count median diameter. The intrapulmonary distribution of particles was analyzed 1 hr or 24 hr after the end of exposure, using energy-filtering transmission electron microscopy for elemental microanalysis of individual particles. In an *in vitro* study, we exposed pulmonary macrophages and red blood cells to fluorescent polystyrene microspheres (1, 0.2, and 0.078 μm) and assessed particle uptake by confocal laser scanning microscopy. Inhaled ultrafine titanium dioxide particles were found on the luminal side of airways and alveoli, in all major lung tissue compartments and cells, and within capillaries. Particle uptake *in vitro* into cells did not occur by any of the expected endocytic processes, but rather by diffusion or adhesive interactions. Particles within cells are not membrane bound and hence have direct access to intracellular proteins, organelles, and DNA, which may greatly enhance their toxic potential.

High concentrations of airborne particles have been associated with increased pulmonary and cardiovascular mortality, with indications of a specific toxicologic role for ultrafine particles (UFPs; particles with diameters < 0.1 μm) (Peters et al. 1997). UFPs may induce inflammatory and prothrombotic responses, promoting atherosclerosis, thrombogenesis, and the occurrence of other cardiovascular events (Schulz et al. 2005). Human data suggest that inhaled UFPs influence lung physiology (Pietropaoli et al. 2004). UPFs may also affect the autonomic nervous system or act directly on cells in various organs and induce mutations (Harder et al. 2005; Samet et al. 2004). After exposure of the respiratory system to UFPs, the UFPs may appear within hours in many compartments of the body, including the liver, heart, and nervous system (Brown et al. 2002; Kreyling et al. 2002; Oberdörster et al. 2004).

UFPs are formed by gas-to-particle conversion or by incomplete fuel combustion. Despite considerable efforts to reduce air pollution, the environmental burden by UFPs may have increased rather than decreased over time (Kreyling et al. 2003). Moreover, the fast-growing nanotechnology industry generates new UFPs daily, which may become aerosolized at some stage and may present additional health risks. UFPs possess increased toxicity compared with larger particles composed of the same materials (Ferin et al. 1992). Their environmental burden is characterized by high number concentrations but low mass concentrations. Thus, a relatively large surface area per unit mass facilitates adsorption of various organic compounds from the ambient air and enhances interaction with biological molecules within the organism.

Deposition of UFPs in the respiratory system is caused by diffusional displacement. Depending on particle size, deposition occurs efficiently in the nose, the conducting airways, and the alveoli. Although particles with diameters > 1 μm usually remain on the epithelial surface upon their deposition (Gehr et al. 1990; Geiser et al. 2003; Schürch et al. 1990) and are subjected to clearance by cough, mucociliary transport, and/or phagocytosis by macrophages, UFPs seem to penetrate the boundary membranes of the lungs rapidly—a unique feature for insoluble particles (Brown et al. 2002; Kreyling et al. 2002; Oberdörster et al. 2002). In addition, transport across the olfactory epithelium and accumulation in the brain were reported for various UFP types (Oberdörster 2004). *In vitro* experiments revealed penetration of UFPs into mitochondria of macrophages and epithelial cells that was associated with oxidative stress and mitochondrial damage (Li et al. 2003).

Because everyone on earth inevitably inhales thousands to millions of UFPs with each breath, it is important to assess health risks by UFP air pollution. The costs of actions to be taken to reduce ambient aerosol particles are high and will affect the economy greatly, presenting an urgent need to clarify the fate of inhaled UFPs. To date, the mechanisms by which UFPs penetrate boundary membranes and the distribution of UFPs within tissue compartments of their primary and secondary target organs are largely unknown.

This study is the first to investigate the distribution of inhaled UFPs within lungs at the individual particle level and combines different experimental approaches—an *in vivo* inhalation study in rats and an *in vitro* cell exposure study on pulmonary macrophages and red blood cells (RBCs).

In the *in vivo* experiments, rats inhaled an ultrafine titanium dioxide aerosol of 22 nm count median diameter (CMD) during 1 hr, resulting in a deposition of 4–5 μg TiO_2_ per animal. The intrapulmonary distribution of deposited particles was analyzed immediately or 24 hr after the end of exposure, using energy-filtering transmission electron microscopy (EFTEM) to allow elemental microanalysis of individual particles (Kapp et al. 2004).

In the *in vitro* study, we exposed cultured porcine pulmonary macrophages and human RBCs to fluorescent polystyrene microspheres with diameters of 1, 0.2, and 0.078 μm and assessed particle uptake by confocal laser scanning microscopy (CLSM).

## Materials and Methods

### Animals.

The animal experiments were conducted under federal guidelines for the use and care of laboratory animals (German Animal Protection Law) and were approved by the District of Upper Bavaria (Approval No. 211-2531-108/99) and by the GSF Institutional Animal Care and Use Committee, as well as in accordance with the Swiss Federal Act on Animal Protection and the Swiss Animal Protection Ordinance. Ten young, adult, male WKY/NCrl BR rats [body weight (bw) 250 ± 10 g (mean ± SD; Charles River, Sulzfeld, Germany] were housed under standard conditions, with access to food and water *ad libitum*, in a room controlled for humidity (55% relative humidity) and temperature (22°C) and with lighting on a 12-hr day/night cycle. Animals were anesthetized by intramuscular injection of a mixture of medetomidine (Domitor; 150 μg/100 g bw; Pfizer GmbH, Karlsruhe, Germany), midazolam (Dormicum; 0.2 mg/100 g bw; Hoffmann-La Roche AG, Grenzach-Wyhlen, Germany), and Fentanyl (0.5 μg/100 g bw; Janssen-Cilag GmbH, Neuss, Germany) for inhalation and lung fixation and anticoagulated by intra-peritoneal injection of 2,000 IU heparin (Heparin-Natrium-ratiopharm, ratiopharm GmbH, Ulm/Donautal, Germany) for lung fixation (Kapp et al. 2004). Anesthesia was antagonized by subcutaneous injection of atipamezole (Antisedan; Pfizer GmbH), flumazenil (Anexate, Hoffmann-La Roche AG), and naloxone (Narcanti, Janssen Animal Health, Neuss, Germany).

### Aerosols and inhalation.

Titanium is suitable for EFTEM because it does not interfere with the heavy metals used for tissue preparation (Kapp et al. 2004). TiO_2_ is inert and nonpathogenic (Templeton 1994), and ultrafine aerosols thereof remain as insoluble particles (Donaldson et al. 2002). Generally, commercially available TiO_2_ particles (Degussa, Düsseldorf, Germany) show a positive Zeta potential of 20–30 mV as measured by Malvern Zetasizer 3000 HS (Malvern Instruments Ltd., Malvern, Worcestershire, UK) The generation and inhalation of the TiO_2_ aerosol used in this study has been described in detail by Kapp et al. (2004). Briefly, ultrafine TiO_2_ aerosols were generated with a Palas spark generator (Palas GmbH, Karlsruhe, Germany) in a pure argon plus 0.1% oxygen stream. The aerosol was quasi-neutralized by a radioactive ^85^Kr source, diluted, and conditioned for inhalation. Particle size distribution and number concentration were monitored continuously by a differential electrical mobility particle sizer and a condensation particle counter. An aerosol of 22 nm CMD (geometric SD of 1.7) was produced. The mean number concentration was 7.3 × 10^6^ particles/cm^3^ (SD 0.5 × 10^6^ particles/cm^3^), corresponding to a mass concentration of 0.11 mg/m^3^. The 22 nm particles measured as aerosol particles are already agglomerates of smaller primary structures formed immediately after spark ignition and condensation. The estimated size of the primary structures was 4 nm as derived from the measured specific surface area of 330 m^2^/g of the UFPs produced in this study and the TiO_2_ bulk density of 4.2 g/cm^3^.

Each anesthetized rat was placed in an airtight plethysmograph box. The animals inhaled the aerosol in pairs (one each for the 1-hr and 24-hr examinations) for 1 hr via an endotracheal tube by negative-pressure ventilation (−1.5 Pa) at a breathing frequency of 45/min, resulting in a minute volume of about 200 cm^3^/min (Kreyling et al. 2002).

From breathing and aerosol parameters, the deposited amount of TiO_2_ was calculated to be 4–5 μg in each rat. After aerosol exposure, anesthesia of one of the paired rats was antagonized as described above, and the animal was returned to its cage for 24 hr.

### Lung fixation and tissue preparation.

Lungs were fixed either 1 hr or 24 hr after the aerosol inhalation by sequential intravascular perfusion with buffered 2.5% glutaraldehyde (Agar Scientific Ltd., Plano GmbH, Wetzlar, Germany), 1.0% osmium tetroxide (Simec, Zofingen, Switzerland), and 0.5% uranyl acetate (Fluka Chemie GmbH, Sigma-Aldrich, Buchs, Switzerland). Lungs were then subjected to systematic tissue sampling, dehydration in a graded series of ethanol, and embedding in Epon (Fluka) (Im Hof et al. 1989; Kapp et al. 2004). Ultrathin (≤50 nm) sections were cut from five to eight tissue blocks per animal, mounted onto uncoated 600-mesh copper grids, and stained with uranyl acetate and lead citrate (Ultrostain, Leica, Glattbrugg, Switzerland).

### *Particle localization and elemental micro-analysis* in situ.

On ultrathin sections, 12 fields, corresponding to an area of 1,820 μm^2^ each, were systematically sub-sampled and investigated for the presence and localization of TiO_2_ particles in a LEO 912 transmission electron microscope (LEO, Oberkochen, Germany) equipped with an in-column energy filter allowing energy dispersion for element specific contrast. TiO_2_ particles were identified by parallel electron energy-loss spectroscopy (parallel-EELS), electron spectroscopic imaging, and image-EELS (Kapp et al. 2004). For elemental micro-analysis, we used the L _2 , 3_ edge of Ti at 456 eV energy loss. We obtained bright-field and structure-sensitive micrographs (recorded at 250 eV), as well as element-specific contrast for TiO_2_, by digital acquisition.

### Cell culture experiments.

Porcine lung macrophages (provided by K. McCullough and H. Gerber, Institute for Virus Diseases and Immune Prophylaxis, Mittelhäusern, Switzerland) were cultured at 10^6^ cells/mL in two-chamber slides (VWR International AG, Dietikon, Switzerland) for 24 hr at 37°C and 5% CO_2_ in RPMI 1640 medium (containing 25 mM Hepes; LabForce AG, Nunningen, Switzerland) with 10% fetal bovine serum (LabForce), 1% l-glutamine (LabForce), and 1% penicillin/streptomycin (Gibco BRL, Invitrogen AG, Basel, Switzerland). Human RBCs, always freshly isolated from the same donor, were cultured at 8 × 10^6^ cells/mL and for 6–24 hr, as described above.

For CLSM, fluorescent polystyrene microspheres with diameters of 1, 0.2, and 0.078 μm (Fluoresbrite plain yellow green; Polysciences, Chemie Brunschwig AG, Basel, Switzerland) were added to the cells at 10^10^ particles/mL in supplement-free RPMI 1640 medium for 4 hr. To inhibit phagocytic uptake, cells were pretreated with cytochalasin D (cytD, 10 μg/mL; Fluka) for 30 min and during incubation with particles (Thiele et al. 2001). Macrophages were then washed in phosphate-buffered saline (PBS), fixed in 3% paraformaldehyde/PBS, treated with 0.1 M glycine/PBS, permeabilized with 0.2% Triton X-100/PBS, and stained for F-actin with rhodamine-phalloidin (1:100; Molecular Probes, VWR International AG, Lucerne, Switzerland) for 60 min at 37°C. RBCs were fixed in 2.5% glutaraldehyde/PBS, resulting in a red autofluorescence. Preparations were mounted in PBS:glycerol (2:1) containing 170 mg/mL Mowiol 4-88 (Calbiochem, VWR International AG).

For TEM analysis, RBCs were incubated with 0.025 μm gold particles (ANAWA Trading SA, Wangen, Switzerland) at 6.6 × 10^10^ particles/mL. RBCs were fixed with buffered 2.5% glutaraldehyde, 1% osmium tetroxide, and 0.5% uranyl acetate, and prepared for TEM as described above for lung tissue.

### Microscopic analysis of cultured cells.

We used a MicroRadiance system from Bio-Rad (Hemel Hempstead, UK) combined with an inverted Nikon microscope (Eclipse TE3000; lasers: GHe/Ne 543 nm and Ar 488 nm) (Nikon, Küsnacht, Switzerland). Optical sections with a voxel dimension of 50 × 50 × 200 nm were taken with a 100× /1.4 plan-apochromate objective. We performed image processing and visualization using IMARIS software (Bitplane AG, Zurich, Switzerland). We applied a deconvolution algorithm using Huygens2 software (Scientific Volume Imaging B.V., Hilversum, Netherlands) to increase axial and lateral resolutions and to decrease noise (Rothen-Rutishauser et al. 1998). Experiments were performed in triplicate or quadruplicate, and 30–50 cells were scanned by CLSM for each data point. We examined ultrathin sections of RBCs incubated with gold particles in a Philips 300 TEM at 60 kV (Philips, Zurich, Switzerland).

## Results

### Distribution of inhaled UFPs in lungs.

**Figure 1:**
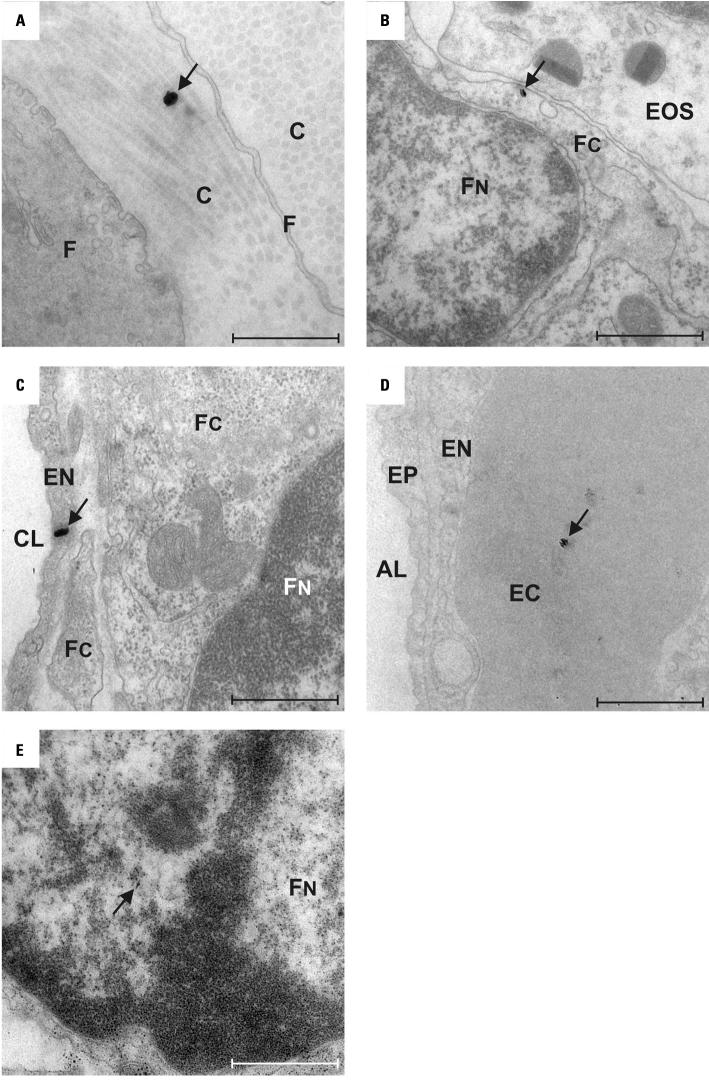
EFTEM micrographs of particles (arrows) in the lung parenchyma. Abbreviations: AL, alveolar lumen; C, collagen fibril; CL, capillary lumen; EC, erythrocyte; EN, capillary endothelial cell; EOS, eosinophil granulocyte; EP, epithelium; F, fibroblast; FC, fibroblast cytoplasm; FN, fibroblast nucleus. (*A*) Particle (diameter, 85 nm) in the connective tissue between Cs. (*B*) Particle (diameter, 41 nm) in the FC near its FN. (*C*) Particle (diameter, 81 nm) in the cytoplasm of an EN. (*D*) Particle (diameter, 41 nm) within an EC in the CL. (*E*) Particle (diameter, 50 nm) within the FN. Bars = 500 nm.

**Figure 2:**
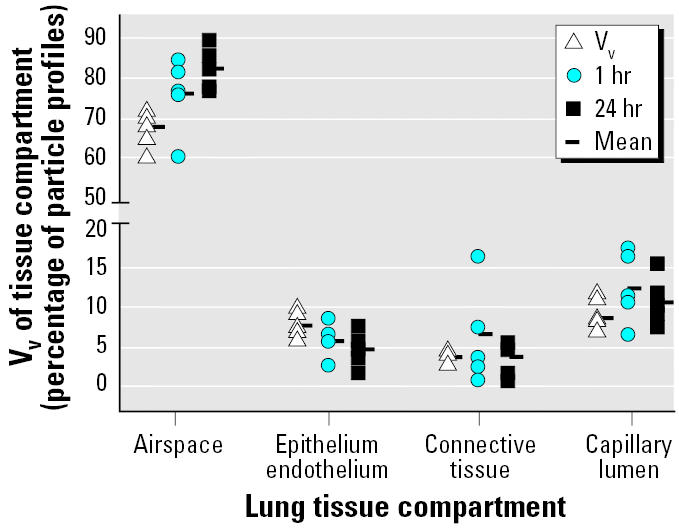
Relative distribution of particles localized in the different lung compartments at 1 hr and 24 hr after inhalation. Volume densities (V_v_) for lung tissue compartments from Burri et al. (1973), Pinkerton et al. (1992), and Tschanz et al. (1995, 2003).

We found TiO_2_ particles on the luminal side of airways and alveoli as well as within each tissue compartment of the lung (Figures 1 and 2). Particles were localized within epithelial and endothelial cells, within fibroblasts and between collagen fibrils in the connective tissue, within blood capillaries, and even within RBCs (Figure 1). Intracellular particles were localized most often in the cytoplasm and rarely within the nucleus. Particles within cells were not membrane bound. On average, 79.3 ± 7.6% (mean ± SD) of the particles were found on the luminal side of airways and alveoli, 4.6 ± 2.5% were within epithelial or endothelial cells, 4.8 ± 4.5% within the connective tissue, and 11.3 ± 3.9% within the capillaries. The relative distributions of particles among different lung compartments at 1 hr and at 24 hr after inhalation were not different from each other and correlated with the volume fractions of the respective compartments (Figure 2).

### Uptake of UFPs by macrophages and RBCs.

**Figure 3:**
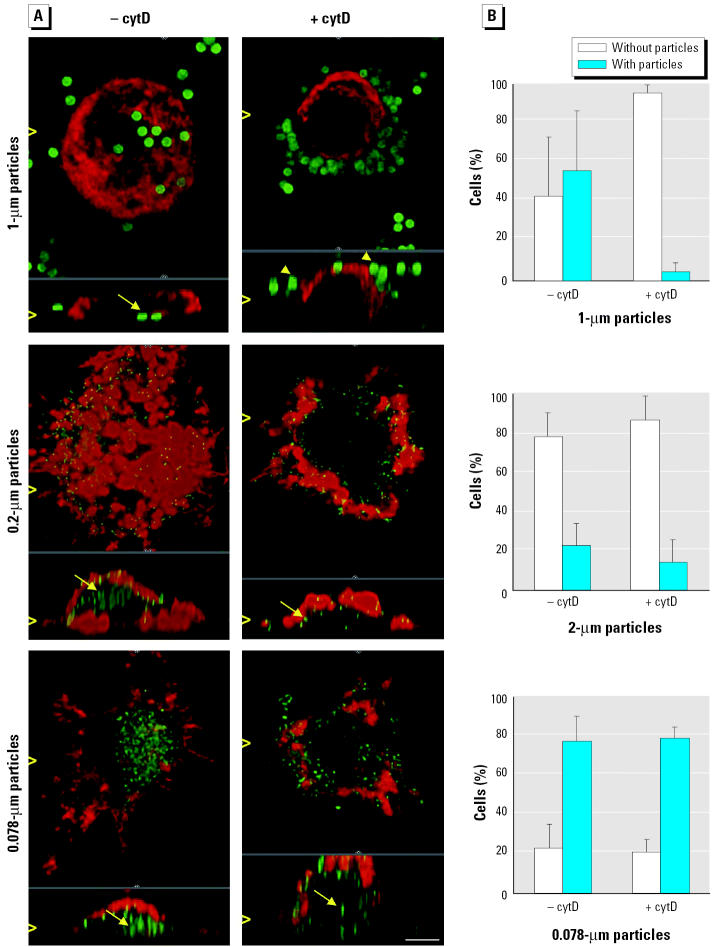
(*A*) CLSM micrographs of fluorescent polystyrene spheres (1, 0.2, and 0.078 μm) taken up by macrophages in the absence (−) or presence (+) of cytD (bar = 2 μm); F-actin is shown in red, and particles are green. The xy and xz projections allow clear differentiation between internalized (arrows) and extracellular (arrowheads) particles; projections are indicated by open arrowheads. (*B*) Particle uptake by macrophages in the absence and presence of cytD. Data are expressed as mean ± SD of three to four experiments scanning 30–50 cells each by CLSM.

**Figure 4:**
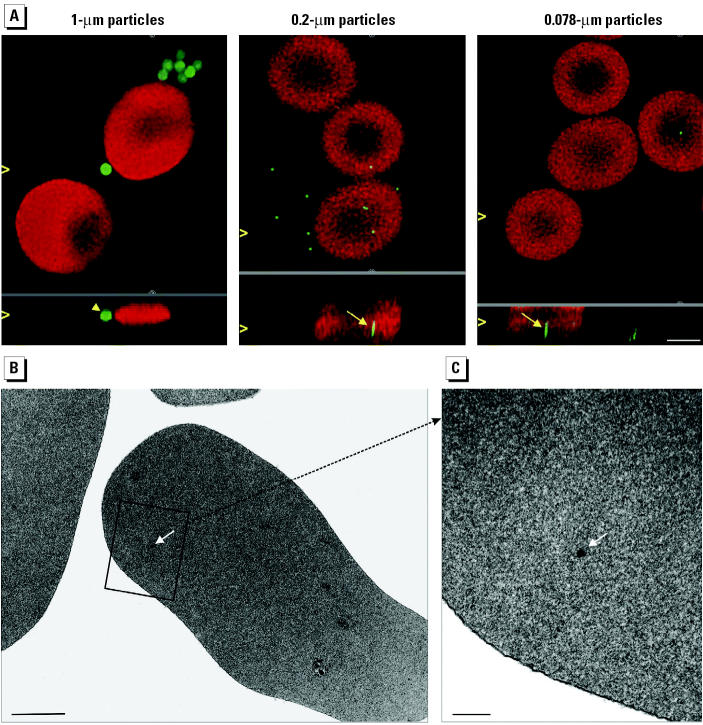
(*A*) CLSM micrographs of fluorescent polystyrene spheres taken up by RBCs. Autofluorescence of the cells is shown in red, and particles are green (bar = 2 μm). The xy and xz projections allow clear differentiation between internalized (arrows) and extracellular (arrowheads) particles; open arrowheads mark the position of the projections. (*B, C*) TEM micrographs showing uptake of 0.025 μm gold particles by RBCs; the particles are not membrane bound (arrows). (*B*) Bar = 1 μm. (*C*) Higher magnification of portion of (*B*); bar = 0.2 μm.

We studied the uptake of fine (1–0.2 μm) and ultrafine (< 0.1 μm) fluorescent microspheres in cultured macrophages as a model for phagocytic cells and in human RBCs as a model for nonphagocytic cells. Additionally, we treated macrophage cultures with cytD to block phagocytosis. Cells were prepared for CLSM, and a deconvolution algorithm was applied to increase lateral as well as axial resolution. We found particles of all sizes within macrophages (Figure 3A), however, with different percentages of cells involved in particle uptake. On average, 77 ± 15% (mean ± SD) of the macrophages contained UFPs, 21 ± 11% contained 0.2 μm particles, and 56 ± 30% contained 1 μm particles (Figure 3B). CytD did not inhibit the uptake of ultrafine and 0.2 μm particles but blocked the uptake of 1 μm particles by these cells (Figure 3B). We found ultrafine and 0.2 μm, but not 1 μm, particles in RBCs (Figure 4A). TEM analysis of RBCs incubated with 0.025 μm gold particles showed that intracellular particles were not membrane bound (Figure 4B).

## Discussion

The ultrastructural analyses of lung tissue demonstrated that 1 hr after aerosol inhalation, 24% of ultrafine TiO_2_ particles, on average, were located within and beyond the epithelial barrier (i.e., in the main lung tissue compartments, in the cytoplasm, and in the nucleus of the cells). These results confirm data from human studies, where the inhalation of ultrafine carbon particles affected pulmonary diffusing capacity (Pietropaoli et al. 2004), suggesting that particles in the interstitium have physiologic effects.

This study also provides evidence for some particle translocation into the micro-vasculature. In previous studies on iridium particles, we found minute fractions of particles translocated into secondary target organs (Kreyling et al. 2002; Semmler et al. 2004b). Different particle materials—iridium versus TiO_2_—may have resulted in different particle translocation patterns, and we do not know the exact composition and structure of the ultrafine particle surface, which is likely to influence particle translocation. The present study focuses on particle distribution within the primary target organ, the lung, and the results do not determine which fraction of particles may have escaped the lung micro-vasculature to be systemically circulated.

Particles found within cells were not membrane bound, indicating a nonendocytic uptake. In addition, the overall distribution pattern of the particles in the lungs (i.e., the percentages of particles in the different lung compartments) at 1 hr and at 24 hr after particle inhalation was the same and was correlated with the volume densities of the corresponding lung compartments, implying that ultrafine TiO_2_ particles can move between tissue compartments without restraint. Our results are in contrast with those obtained by Stearns et al. (2001), who studied the uptake of ultrafine TiO_2_ particles *in vitro* in the A549 epithelial cell line. They found that membrane-bound vesicles contained mostly large aggregates of TiO_2_. Sometimes vesicles with clusters consisting of as few as two to three particle profiles were observed. In a pilot study *in vitro* with porcine macrophages, we obtained similar results (data not shown). Whether these clusters contained few particles only or were sectioned through the top of larger ones is not known. However, because ultrafine TiO_2_ aggregate very quickly in polar liquids such as cell culture medium and because particle concentration was fairly high, as seen from micrographs, it is very likely that particles aggregated within the cell culture medium and that cells engulfed these large clusters by an endocytic pathway. Particle agglomeration on the lung epithelium during the 1-hr inhalation, however, is very unlikely because of the size of the inner lung surface and the number of deposited UFPs.

The fact that 80% of the retained TiO_2_ particles were still on the luminal side of the epithelium even 24 hr after inhalation is surprising and in contrast to previous studies on the lavageability of ultrafine iridium particles, where only 20% of the particles could be lavaged from the epithelial surfaces 24 hr after inhalation (Kreyling et al. 2002). The low lavageability of ultrafine iridium particles in contrast to high lavageability of 80% of 0.5–10-μm particles (Oberdörster et al. 2002) was interpreted as either higher adhesion of UFPs to epithelial membranes or epithelial uptake and penetration of UFPs into the interstitium. The large fraction of TiO_2_ particles on the luminal side of the epithelium in the present study strongly supports higher adhesion of UFPs to epithelial structures. However, it must also be considered that proteins are very likely to bind rapidly to the particles, which then may affect the further metabolic fate of the particles in terms of their adhesion, residence time on the epithelium or uptake, and even penetration through the epithelium. Along this line, the difference of the two particle materials and surfaces may have led to binding of those proteins, which then may have mediated major uptake and penetration of iridium particles into and through the epithelium. We already have first evidence that ultrafine commercial TiO_2_ particles bind more readily to other proteins in the lung-lining fluid than carbonaceous and amorphous silica particles (Semmler et al. 2004a).

Microscopic analyses of phagocytic and nonphagocytic cells incubated with different particle types showed that macrophages take up fine and ultrafine polystyrene microspheres and that treatment with cytD inhibits the uptake of 1.0-μm particles, but not uptake of the smaller particles, by these cells. Ultrafine polystyrene and gold particles also entered RBCs and were not membrane bound.

The mechanisms of intracellular uptake of macromolecules, particles, and even cells are subsumed as endocytosis. Material to be ingested is progressively enclosed by the plasma membrane, which eventually detaches to form an endocytic vesicle. Phagocytosis and pinocytosis are distinguished by the size of endocytic vesicles formed. Phagocytosis, a receptor-mediated, actin-based process, is characteristic for neutrophils, macrophages, and dendritic cells. It is the main mechanism for the clearance of insoluble 1- to 3-μm particles from the alveoli. Pinocytosis involves the ingestion of fluid and solutes via vesicles of about 100 nm in diameter. There are at least four basic mechanisms, most of which can be demonstrated in lungs and involve specific receptor–ligand interactions: *a*) macro-pinocytosis, *b*) clathrin-mediated, actin-based endocytosis, *c*) caveolae-mediated endo- or transcytosis, and *d*) clathrin- and caveolae-independent endocytosis (Conner and Schmid 2003). Kruth et al. (1999) described an additional endocytic process, patocytosis, in which hydrophobic polystyrene particles < 0.5 μm are transported through induced plasma membrane channels into an extensive labyrinth of interconnected membrane-bound compartments. None of these endocytic pathways, all of which include vesicle formation, is likely to account for the translocation of UFPs in our study, as intracellularly localized particles were not membrane bound. Moreover, because RBCs contained UFPs and cytD treatment of macrophages did not prevent UFP translocation into these cells, particle uptake by any actin-based mechanism can also be excluded.

Transport via pores, as suggested for lung–blood substance exchange (Conhaim et al. 1988; Hermans and Bernard 1999), is another potential mechanism for UFP translocation. TiO_2_ particles may diffuse through such pores. A transport mechanism by diffusion is consistent with the observed spatial distribution of UFPs in our inhalation study. Thus far, signal-mediated transport via pores has been demonstrated only for ultrafine gold particles of up to 39 nm in diameter, through the nuclear pore complex in *Xenopus* oocytes, where transport velocities depended on particle size (Panté and Kann 2002).

Passive uptake (not triggered by receptor–ligand interactions) may also occur by electrostatic, Van der Waals, or steric interactions, subsumed under “adhesive interactions” (Rimai et al. 2000). Rimai et al. showed that 8-μm glass particles were approximately 90% engulfed by a polystyrene substrate, compared with 22-μm particles, which were only 30% engulfed. However, the influence of particle size on their engulfment was not clarified in these publications.

Several concepts for the nonspecific engulfment of particles through interfacial structures (including cell membranes) have been suggested. A thermodynamic model using the “wettability criterion” was successful in predicting passive particle uptake, although it did not take into account the elastic properties of the cell membrane (Chen et al. 1997). In another thermodynamic analysis combined with a molecular dynamics simulation, Bresme and Quirke (1999) showed that line tension influences the wetting behavior of nanoparticles at liquid–vapor and liquid–liquid interfaces. These authors found negative line tension values for particles of a few nanometers in diameter, but positive values for those an order of magnitude larger. A negative line tension favors the initial wetting of a spherical particle after its approach to an interface.

Shanahan (1990) studied the engulfment of solid-surface heterogeneities equivalent to particles in the nanometer range by unbalanced capillary forces (free energy perturbations). Thermal capillary waves cause fluid droplets to coalesce with a fluid substrate by film drainage at the interface, breakage of the film, and intrusion of the particle into the bulk phase (Aarts et al. 2004). Thus, thermal capillary fluctuations may enhance particle transport through cell membranes.

Experimental results demonstrate consistently greater immersion of smaller particles than larger ones into a liquid substrate covered by surfactant film *in vitro* as well as *in situ* in airways. These results support the concept that line tension plays a significant role in particle displacement (Geiser et al. 2000; Schürch et al. 1999).

It remains to be determined which chemical and physical properties of membranes and particles are responsible for the translocation of UFPs *in vivo*. Interestingly, we did not see any difference in particle uptake *in vitro* with respect to differing surface charges or surface chemistry when we used three different particle types: a metal, a metal oxide, and a synthetic polymeric material (data not shown). However, these particles were added to the cells in suspension and did not approach the cells from the air or require passage through a surfactant film first, as in the *in vivo* inhalation experiments. In these *in vivo* experiments, electrostatic interactions are likely important for particle deposition and subsequent retention.

In summary, UFPs of various materials can cross any cellular membrane, but neither endocytosis, which is based on vesicle formation, nor any actin-based mechanisms are likely to account for UFP translocation into the cell. Our results from the inhalation experiments with TiO_2_ particles point to a transport mechanism that includes adhesive interactions or, in terms of thermodynamics, interfacial and line tension effects. In addition, particle diffusion and uptake promoted by thermal capillary waves might play a role in particle transport through membranes. After the deposition of nanometer-size particles, their further fate may be largely independent from particle surface chemistry and charge.

Consequently, it is a possible fate of inhaled ambient UFPs that they are transferred from the lungs to most other organs. In a first analysis of ultrathin sections from hearts of the same rats, we found ultrafine TiO_2_ particles in the connective tissue, that is, within fibroblasts (data not shown). There may be no means on the cellular level to prevent, influence, or direct their uptake. Moreover, the toxic potential of UFPs is greatly enhanced by their free location and movement within cells, which promote interactions with intracellular proteins and organelles and even the nuclear DNA.

Potential health implications of our findings are related not only to ambient UFPs but also to engineered “nanoscaled particles,” which may be released into our environment during their production, transport, and aging, or during waste disposal (The Royal Society 2004). Routes of exposure to nanomaterials include oral, cutaneous, and inhalative uptake, the latter being addressed in the present study. Although the number of particles translocated into the cells may vary substantially (Nanosafe 2004) according to their physicochemical properties, the data of the present study strongly suggest that adverse health outcomes associated with the uncontrolled presence of nanoscale particles in tissues require further attention.
